# *In vivo* Effects of Romidepsin on T-Cell Activation, Apoptosis and Function in the BCN02 HIV-1 Kick&Kill Clinical Trial

**DOI:** 10.3389/fimmu.2020.00418

**Published:** 2020-03-20

**Authors:** Miriam Rosás-Umbert, Marta Ruiz-Riol, Marco A. Fernández, Marta Marszalek, Pep Coll, Christian Manzardo, Samandhy Cedeño, José M. Miró, Bonaventura Clotet, Tomáš Hanke, José Moltó, Beatriz Mothe, Christian Brander, the BCN02 study group, Susana Benet

**Affiliations:** Author Affiliations: IrsiCaixa AIDS Research Institute-HIVACAT, Hospital Universitari Germans Trias i Pujol, Badalona, Spain; Fundació Lluita contra la Sida, Hospital Universitari Germans Trias i Pujol, Badalona, Spain; Germans Trias i Pujol Research Institute, Badalona, Spain; Pharmacokinetic/pharmacodynamic modeling and simultation, Institut de Recerca de l'Hospital de la Santa Creu i Sant Pau-IIB Sant Pau, Barcelona, Spain; Hospital Clinic-HIVACAT, IDIBAPS, University of Barcelona, Spain; (currently working at Hospital Universitari Arnau de Vilanova, Lleida, Spain); Projecte dels noms, BCN Checkpoint, Barcelona, Spain; The Jenner Institute, The Nuffield Department of Medicine, University of Oxford, UK; ^1^IrsiCaixa AIDS Research Institute-HIVACAT, Badalona, Spain; ^2^Department of Cellular Biology, Physiology and Immunology, Universitat Autònoma de Barcelona (UAB), Barcelona, Spain; ^3^Flow Cytometry Facility, Health Sciences Research Institute Germans Trias i Pujol, Badalona, Spain; ^4^Hospital Clinic- IDIBAPS, University of Barcelona, Barcelona, Spain; ^5^Fundació Lluita contra la Sida, Hospital Universitari Germans Trias i Pujol, Badalona, Spain; ^6^Department of Infectious Diseases, Hospital Germans Trias i Pujol, Badalona, Spain; ^7^Centre for Health and Social Care Research (CESS), Faculty of Medicine, University of Vic – Central University of Catalonia (UVic – UCC), Vic, Spain; ^8^The Jenner Institute, University of Oxford, Oxford, United Kingdom; ^9^Joint Research Center for Human Retrovirus Infection, Kumamoto University, Kumamoto, Japan; ^10^ICREA, Pg. Lluis Companys, Barcelona, Spain

**Keywords:** romidepsin, HDAC inhibitor, kick&kill strategy, therapeutic vaccine, latency reversing agent (LRA)

## Abstract

Romidepsin (RMD) is a well-characterized histone deacetylase inhibitor approved for the treatment of cutaneous T-cell lymphoma. *in vitro* and *in vivo* studies have demonstrated that it is able to induce HIV-1 gene expression in latently infected CD4^+^ T cells from HIV-1^+^ individuals on suppressive antiretroviral therapy. However, *in vitro* experiments suggested that RMD could also impair T-cell functionality, particularly of activated T cells. Thus, the usefulness of RMD in HIV-1 kick&kill strategies, that aim to enhance the immune system elimination of infected cells after inducing HIV-1 viral reactivation, may be limited. In order to address whether the *in vitro* observations are replicated *in vivo*, we determined the effects of RMD on the total and HIV-1-specific T-cell populations in longitudinal samples from the BCN02 kick&kill clinical trial (NCT02616874). BCN02 was a proof-of-concept study in 15 early treated HIV-1^+^ individuals that combined MVA.HIVconsv vaccination with three weekly infusions of RMD given as a latency reversing agent. Our results show that RMD induced a transient increase in the frequency of apoptotic T cells and an enhanced activation of vaccine-induced T cells. Although RMD reduced the number of vaccine-elicited T cells secreting multiple cytokines, viral suppressive capacity of CD8^+^ T cells was preserved over the RMD treatment. These observations have important implications for the design of effective kick&kill strategies for the HIV-1 cure.

## Introduction

Current antiretroviral therapy (ART) effectively suppresses HIV-1 replication in plasma, but it is not able to completely eliminate the virus from infected individuals. Cessation of antiretroviral treatment results in a rebound of plasma viremia within 3–4 weeks in most individuals (1). This rapid viral rebound after treatment interruption is due to the existence of a latent viral reservoir and the inability of the immune system to effectively contain viral replication. To date, numerous strategies have been pursued to achieve a functional cure or virus eradication, including early ART initiation, ART intensification (2–6), passive administration of antibodies (7), therapeutic vaccination (8–13) and gene therapy (14, 15), among others.

HIV-1 kick&kill strategies are based on the use of latency reversing agents (LRA) to induce production of HIV-1 proteins in latently infected cells and render these cells susceptible to vaccine-induced virus-specific cytolytic T lymphocytes (CTL). The interest in LRA able to reactivate the latent provirus has increased over the past decade, with histone deacetylation inhibitors (HDACi) being some of the best characterized agents both *in vitro* and *in vivo*. The inhibitory effect of HDACi on histone deacetylation results in a higher degree of acetylated histones, causing opening of chromosomes and increased gene transcription. In *ex vivo* isolated cells from ART-suppressed HIV-1-infected individuals, exposure to HDACi resulted in reactivation of the integrated HIV-1 and led to viral protein expression by latently infected cells (16–18). Some HDACi, such as vorinostat (SAHA), panobinostat, and romidepsin (RMD) have also been tested for their *in vivo* potential to reverse HIV-1 latency (19–22). RMD, a cyclic depsipeptide naturally produced by *Chromobacterium violaceum*, is a pan-HDACi that inhibits class I HDACs. RMD was clinically developed as an anti-cancer drug and is approved for the treatment of cutaneous T-cell lymphoma (23). Furthermore, RMD has been shown to induce HIV-1 gene expression in latently infected cells *in vitro* (18, 24) and *in vivo*, when administrated alone (22) and in combination with therapeutic vaccine Vacc-4x (25) in chronically-infected ART-suppressed individuals. Although it was first thought that reactivation of the virus itself would lead to robust immune activation and control of the rebounding virus, it is generally accepted that prior stimulation of the immune effector response by a therapeutic vaccine and/or an immune checkpoint inhibitor, may be needed in order to efficiently eliminate infected cells after LRA exposure (26). Therefore, different immunotherapies are being investigated together with LRAs to test their combined effect, especially combination treatments that include T-cell vaccines.

**Figure 1:**
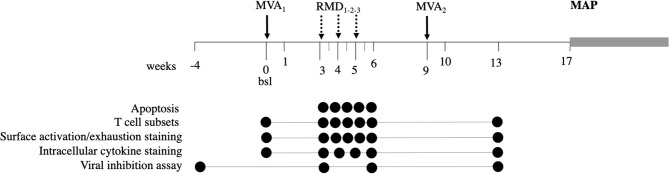
Study design. The BCN02 study was a single arm, open label, proof-of-concept study to address safety and effect on the viral reservoir of a kick&kill strategy combining MVA.HIVconsv vaccines with the HDACi RMD. Timepoints used for the analysis presented here are indicated for each assay by filled circles.

The proof-of-concept BCN02 trial evaluated a kick&kill strategy that combined the HIVconsv T-cell vaccines with the HDACi RMD in a cohort of early-treated, HIV-1- infected individuals. Fifteen participants of BCN01 (12), who previously received simian adenovirus-vectored vaccine ChAdV63.HIVconsv and MVA.HIVconsv, were invited 2–3 years later to receive two more dosing of the MVA.HIVconsv vaccine before (MVA_1_) and after (MVA_2_) three weekly-doses of RMD (RMD_1−2−3_) followed by a monitored antiretroviral pause (MAP) for a period of 32 weeks (NCT02616874). The combined strategy was proven to be safe and vaccination was highly immunogenic. RMD treatment resulted in marked increases in histone acetylation and cell-associated HIV-1 RNA levels compatible with induction of viral transcription. However, the ultimate reduction of the viral reservoir in the BCN02 trial was overall minimal (27). Aside from effects on virus reactivation, for a successful purge of the viral reservoir, it is critical that the LRA used in such strategies does not have any detrimental effects on the vaccine-induced immune cells (28, 29). Here, we assessed the *in vivo* impact of three weekly RMD doses on total and vaccine-induced T cells in longitudinal samples from the BCN02 trial (Figure 1).

## Materials and Methods

### Study and Samples

The BCN02 clinical trial (NCT02616874) was a phase I, open-label, single-arm, multicenter study in Spain (27). The study was approved by the institutional ethical review board of the participating institutions (Reference Nr AC-15-108-R) and by the Spanish Regulatory Authorities (EudraCT 2015-002300-84) and was conducted in accordance with the principles of the Helsinki Declaration and local personal data protection law (LOPD 15/1999). Fifteen participants were immunized with MVA.HIVconsv (MVA_1_, 2 × 10^8^ pfu intramuscularly), followed by three weekly-doses of romidepsin (RMD_1−2−3_, 5 mg/m^2^ body-surface area; BSA) and a second MVA.HIVconsv boost vaccination (MVA_2_, 2 × 10^8^ pfu i.m.) before undergoing a monitored antiretroviral pause (MAP) 8 weeks later and for a maximum of 32 weeks. Cryopreserved peripheral blood mononuclear cells (PBMC) were stored before, at the end and after 8, 24 h (only for RMD_1_), and 3 and 7 days after all RMD doses for immunological and virological studies.

### Flow Cytometry

#### Apoptosis Measurement

PBMC viability was measured using a Pacific Blue™ Annexin V Apoptosis Detection Kit with 7-AAD (BioLegend). Lineage surface markers (CD3, CD4, and CD8) and activation markers (HLA-DR, CD25, and CD69) were included in the staining.

Briefly, 1 × 10^6^ of isolated PBMC were washed in PBS with 1% FBS and resuspend in 100 μl of surface staining solution (CD3, CD4, CD8, CD25, CD69, HLA-DR) and incubated for 20 min. After 2 washes with 300 μl of PBS with 1% FBS, cells were resuspended in 100 μl of Annexin V Binding Buffer with the corresponding Annexin V and 7-AAD. After 15 min of incubation, 250 μl of Binding Buffer was added to each tube and acquired on a LSRII BD cytometer. The percentages of apoptotic and live cells were analyzed using FlowJo software. The gating strategy is summarized in Supplementary Figure 1.

#### T Cells and HIVconsv-Specific T-Cell Lineage, Activation and Cytokine Detection

PBMCs were thawed and stimulated with anti-CD49d and anti-CD28 antibodies (BD) in presence/absence of three peptides pools (containing 58, 54, and 54 peptides) covering the HIVconsv immunogen protein in the presence of GolgiStop for 5 h. Cultures were then stored overnight at 4°C until staining. Cells were stained first with a viability stain (Aqua Live/Dead Fixable Dead Cell Stain kit, Invitrogen), followed by T cell lineage and maduration/activation markers (using anti-CD3-APC Cy7, anti-CD4 PECy5; anti-CD8 PerCP, anti-CCR7 B711, anti-CD45RA BV785, anti-HLA-DR BV650, anti-PD-1 BV605, anti-CD69 APC, and anti-CD25 PEDazzle594 chromogen-conjugated monoclonal antiobodies; BioLegend) and dump channel (using anti-CD19-V450 for B-cells and anti-CD14-V450 mAbs for monocytes; BioLegend) surface staining. Following the fixation and permeabilization step (Fix and Perm kit, Invitrogen), intracellular staining with conjugated antibodies specific for cytokines (IFN-γ A700; Invitrogen, IL-2 PECy7, TNF-α FITC; BiolLegend and MIP1-β PE; RD Systems) was performed. Approximately 10^5^ cells were acquired on an LSRFortessa BD instrument, and analysis was performed using FlowJo 10 software. The gating strategy is summarized in Supplementary Figure 2.

Intracellular cytokine staining analyses were done applying boolean gates in FlowJo 10, subtracting unstimulated signals using Pestle v1.7 program and represented using SPICE v5.35 software (provided by the National Institute of Health, Mario Roeder, ImmunoTechnology Section, Vaccine Research Center, NIAID, NIH, Bethesda) (30).

### Viral Inhibition Assay

CD8^+^ T-cell mediated viral inhibition capacity was measured at 1:1 and 1:10 CD8 effector to CD4 target ratios. Cryopreserved PBMCs were obtained from timepoints before the BCN02 intervention and CD8^+^ cells were depleted by magnetic bead separation (MACS Milteny Biotec). CD8^+^-depleted cells (CD4^+^-enriched fraction) were stimulated with PHA (5 μg/ml) in RPMI plus 10% fetal bovine serum (R10) and antibiotics (penicillin 100 U/mL and streptavidin 100 μg/ml). After 3 days of stimulation, the CD4-enriched fraction was infected by spinoculation with HIV-1_BaL_ and HIV-1_IIIB_ laboratory-adapted strains at a multiplicity of infection (MOI) of 0.01 as reported previously (12, 31). HIV-infected cells were cultured in duplicates or triplicates in R10 medium with 20 U/ml of IL-2 in 96-well round-bottomed plates, alone or together with unstimulated CD8^+^ T cells obtained by positive magnetic bead separation the same day from an additional vial of frozen PBMC from screening (week −4), 3 weeks after MVA_1_ (week 3, postMVA_1_), 1 week after RMD_3_ (week 6, postRMD_3_), 4 weeks after MVA_2_ (week 13, postMVA_2_) timepoints. Cultures at different CD8:CD4 ratios (E:T = 1:1 and 1:10) were harvested after 6 days. Cells were stained first with Aqua Live/Dead stained for surface markers (CD3 APC-H7, BD Biosciences, CD4 PerCP, BD Biosciences, and CD8 APC, BD Biosciences), then permeabilized (FIX & PERM® Cell Permeabilization Kit, ThermoFisher). Cells were then fixed (FIX & PERM® Cell Permeabilization Kit, ThermoFisher) at room temperature and finally stained with anti-Gag p24 antibody (KC-57-FITC; Beckman Coulter). CD8^+^ T-cell antiviral activity is expressed as % inhibition = [(fraction of p24^+^ cells in CD4^+^ T cells cultured alone)–(fraction of p24^+^ cells in CD4^+^ T cells cultured with CD8^+^ T cells)]/(fraction of p24^+^ in CD4^+^ T cells cultured alone) × 100. At least 100,000 cells were collected on a LSRII BD cytometer and analysis was performed using FlowJo 10 software.

### Statistical Analysis

GraphPad Prism version 7 for Windows (San Diego, CA) was used for statistical analysis. Mann-Whitney test and Wilcoxon matched paired test were used for unpaired and paired comparisons, respectively. Significant values were considered for *p* < 0.05.

## Results

### Increased Apoptosis After RMD Exposure

**Figure 2:**
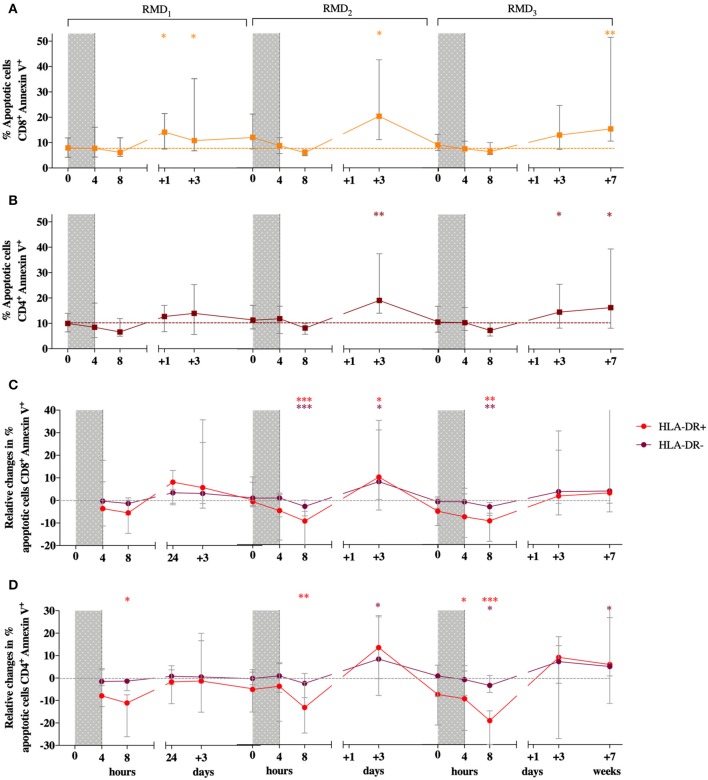
Effect of RMD on the viability on T cells. Apoptotic cells (Annexin V^+^/7AAD^+^ and Annexin V^+^/7AAD^−^) percentages are shown for CD8^+^ **(A)** and CD4^+^ T cells **(B)**. Changes relative to baseline in the percentage of apoptosis in HLA-DR^+^ (in red) and HLA-DR^−^ cells (in purple) are shown for CD8^+^ **(C)** and CD4^+^ **(D)** T cells. RMD administration cycles are indicated by gray bars. Sampling time points at 4 h, 8 h, 3 days, and 7 days after each RMD administration are indicated. Median with interquartile range is shown. *P*-values (*p < 0.05, **p < 0.01, ***p <0.001) are indicative for the corresponding timepoint compared to pre-RMD_1_.

Since RMD was previously described to have a toxic effect *in vitro* (28, 29), especially on activated T cells, we first measured the effect of RMD on cell viability both in total CD8^+^ and CD4^+^ T cells and in activated, HLA-DR^+^ expressing cells in PBMC from the BCN02 participants. Viability in total T cells was assessed by flow cytometry by Annexin V/7AAD staining before and after the three RMD doses. Increases in the number of apoptotic CD8^+^ T cells (Annexin V^+^) were detected at 24 h after RMD_1_ (Wilcoxon signed-rank, *p* = 0.0151) and 3 days after each RMD dose (Wilcoxon signed-rank, RMD_1_ *p* = 0.0413, RMD_2_ *p* = 0.0181, and RMD_3_ *p* = 0.0833, respectively, Figure 2A). A similar pattern was observed in CD4^+^ T cells, with apoptotic cells being significantly increased 3 days after RMD_2_ and RMD_3_ (Wilcoxon signed-rank, RMD_2_ *p* = 0.0067 and RMD_3_ *p* = 0.0413, respectively, Figure 2B). One week after RMD_3_ the levels of apoptotic cells in both CD8^+^ T cells and CD4^+^ were not restored to pre-RMD levels (*p* = 0.0054 for CD8^+^ and *p* = 0.0181 for CD4^+^), suggestive of an accumulative toxic effect of RMD.

As activated T cell have been found to be particularly sensitive to RMD exposure (28), levels of apoptosis in CD4^+^ and CD8^+^ T cell with high or low expression of the activation marker HLA-DR were assessed. Changes in the percentage of apoptotic cells relative to baseline during RMD exposure was comparable between HLA-DR^+^ and HLA-DR^−^ CD8 T cells (Figures 2C,D). The same results were observed in CD4+ T cells. Therefore, non- activated HLA-DR^−^ cells had the same susceptibility to induction of apoptosis due to RMD exposure as the activated HLA-DR^+^ cells.

### CD8^+^ and CD4^+^ Naïve Populations Increase After MVA_1_ and Up to 24 h After 1st Romidepsin Exposure Followed by a Shift to Memory Phenotypes

**Figure 3:**
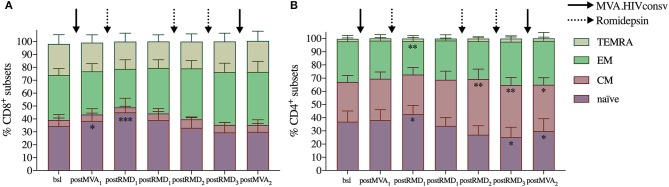
Evolution of T-cell subsets over the course of the intervention. Averages of relative frequency of CD8^+^ **(A)** and CD4^+^ **(B)** T-cell subsets based on CCR7 and CD45RA expression are shown. TEMRA: highly differentiated effector cells expressing CD45RA (CCR7^−^/CD45RA^+^), EM: effector memory cells (CCR7^−^/CD45RA^−^), CM: central memory (CCR7^+^/CD45RA^−^), and naïve (CCR7^+^/CD45RA^+^). *P*-values (*p < 0.05, **p < 0.01, ***p < 0.001) are indicative for the corresponding timepoint compared with values from baseline samples prior to MVA_1_.

As activation status did not impact susceptibility of T cells to RMD-induced apoptosis, we next assessed whether RMD could affect the distribution of different T-cell differentiation subsets in the peripheral blood. Flow cytometry was used to measure the frequency of CD4^+^ and CD8^+^ differentiation subsets defined by CCR7 and CD45RA expression at each timepoint. Three weeks after MVA_1_, the frequency of naïve CD8^+^ T cells increased from median frequency of 30.5–35.2% and up to 49.6% 24 h after RMD_1_ (Wilcoxon signed-rank *p* = 0.0122 and *p* = 0.0005, respectively, Figure 3A). Similar results were observed for naïve CD4^+^ T cells, which increased from 33% at baseline to 35.2% 3 weeks after MVA_1_ and up to 41.7% at 24 h after RMD_1_ (Wilcoxon signed-rank *p* = 0.0227 and *p* = 0.0110, respectively, Figure 3B). The frequency of CD8^+^ and CD4^+^ naïve cells were not further increased after RMD_2_ or RMD_3_, as their frequency actually decreased after RMD_2_ and RMD_3_. At the same time, the median frequency of CD8^+^ effector memory and CD4^+^ central memory T cells progressively increased over RMD_1−2−3_. T-cell differentiation subsets were not further changed 4 weeks after MVA_2_ compared to subset population observed after RMD doses.

### RMD Treatment Increases T-Cell Activation Especially of the HIVconsv-Specific T Cells

**Figure 4:**
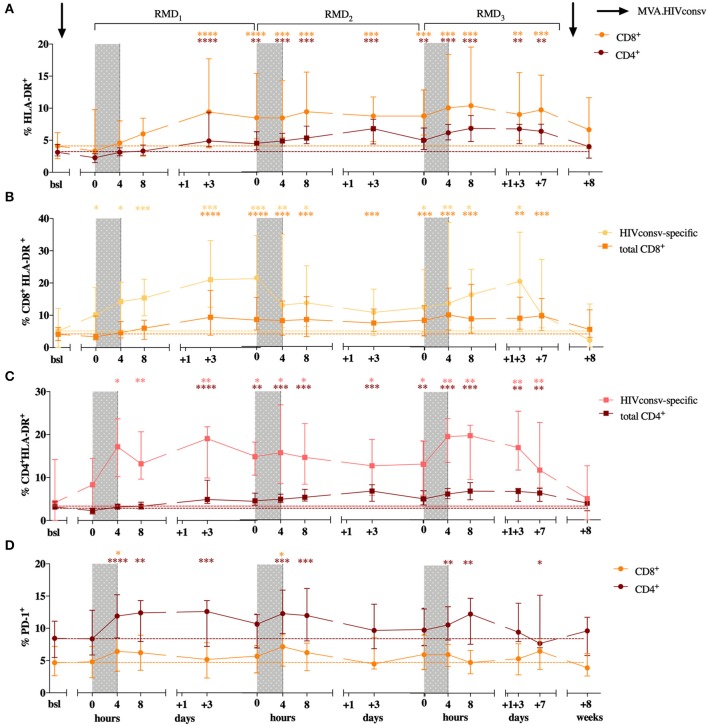
CD4^+^ and CD8^+^ T cell activation status over the course of the intervention. Percentage of HLA-DR expression is shown for CD8^+^ in orange and CD4^+^ T cells in purple **(A)**. Levels of HLA-DR are shown for CD8^+^ **(B)** and CD4^+^ T cells **(C)** in HIVconsv specific and total T cells. HIVconsv-specific T cells were defined upon *in vitro* antigen-specific stimulation and detection based on their ability to produce cytokines (IL-2, MIP1-β, TNF-α, IFN-γ) in response to stimulation. Percentage of PD-1 is shown for CD8^+^ in orange and CD4^+^ T cells in purple **(D)**. Median with interquartile range is shown. *P*-values (*p < 0.05, **p < 0.01, ***p < 0.001, ****p < 0.0001) are indicative for the corresponding timepoint compared with baseline.

In order to assess the effect of RMD on T-cell activation and exhaustion markers, we evaluated the frequency of CD4^+^ and CD8^+^ T cells expressing HLA-DR, CD69, CD25, and PD-1. A peak in the frequency of CD8^+^HLA-DR^+^ T cells was observed 3 days after RMD_1_ (Wilcoxon signed-rank, *p* < 0.0001) and increased levels were maintained over the course of the 3 RMD doses, as their median frequency more than doubled (4.1% at baseline to 9.6% 1 week after RMD_3_, Wilcoxon signed-rank, *p* = 0.0002, Figure 4A). The same results were observed in CD4^+^ T cells, with levels of HLA-DR expression increasing 3 days after RMD_1_ (Wilcoxon signed-rank, *p* < 0.0001) and with twice as many HLA-DR expressing cells at day 7 after RMD_3_ (median 3.1% at baseline to 6.3%, Wilcoxon signed-rank, *p* = 0.0017, Figure 4A) compared to baseline. The largest increase in percentage of cells expressing HLA-DR in both CD4^+^ and CD8^+^ T cells was observed in effector memory (EM) and highly differentiated effector cells expressing CD45RA (TEMRA) (Supplementary Figure 3).

Importantly, upon *in vitro* stimulation with peptides derived from the vaccine immunogen, changes in the HLA-DR^+^ in expression were already detected after MVA_1_. Both, HIVconsv-specific CD8^+^ and CD4^+^ T cells, defined upon *in vitro* antigen-specific stimulation and detection based on their ability to produce cytokines (IL-2, MIP1-β, TNF-α, IFN-γ) in response to antigen specific stimulation, showed detectable increases of HLA-DR^+^ expression earlier and up to higher levels compared to the changes seen in total CD8^+^ and CD4^+^ T cells (Figure 4A). The percentage of HLA-DR^+^ among HIVconsv-specific CD8^+^ cells increased from median of 5.1% at baseline to 10.11% 3 weeks after MVA_1_, and up to 21% 3 days after RMD_1_ (Wilcoxon signed-rank *p* = 0.0245 and *p* = 0.0002, Figure 4B) and percentage of HLA-DR^+^, HIVconsv-specific CD4^+^ cells increased from median of 4.2% at baseline to 8.3% 3 weeks after MVA_1_, and up to 21.8% 3 days after RMD_1_ (Wilcoxon signed-rank *p* = 0.5614 and *p* = 0.0023, Figure 4C). High levels of activation were maintained during all RMD doses (Figures 4C,D) and reached the peak 3 days after RMD_3_ suggestive of an additive effect of RMD on the activation of vaccine-stimulated CD4^+^ and CD8^+^ T cells.

As changes in *in vivo* histone acetylation and induction of viral transcription occur rapidly after RMD exposure (22, 27), we assessed the expression of early activation markers CD69 and CD25 in CD4^+^ and CD8^+^ T cells. Increases in the expression of CD69 in CD4^+^ T cells were observed upon each RMD dosing, with significant increases seen after RMD_2_ and RMD_3_ in the CM subset (from baseline levels of 2.5 to 7.1% (RMD_2_ *p* = 0.0107) and 6.7% (RMD_3_ *p* = 0.0479) and in the TEMRA subset (baseline levels of 2.7 to 9% (RMD_2_ *p* = 0.0203) and 6.6% (RMD_3_ *p* = 0.0315), respectively (Supplementary Figure 4B). As with HLA-DR surface staining, expression of CD69 returned to baseline levels 4 weeks after last vaccination. No significant changes were observed in CD69 expression on CD8^+^ T cells (Supplementary Figure 4A) nor in the expression of CD25 on CD8^+^ or CD4^+^ T cells over the course of the intervention.

The expression of PD-1^+^ increased rapidly in CD8^+^ T cells from median baseline levels of 4.6–6.4, 7.2, and 6.1% at 4 h after the end of RMD_1−2−3_ treatment (Wilcoxon singed-rank, *p* = 0.0215, *p* = 0.0238, and *p* = 0.0574, respectively, Figure 4D). The same kinetics was observed in CD4^+^ T cells where the frequency of PD-1 expressing cells raised from median baseline levels of 8.5–11.9, 12.35, and 10.7% at 4 h after RMD_1−2−3_ (Wilcoxon singed-rank, *p* = 0.0004, *p* = 0.0002, and *p* = 0.0046, respectively, Figure 4D). However, in contrast to the additive effect of RMD_1−2−3_ on T-cell activation markers, changes in PD-1 expression in both CD8^+^ and CD4^+^ T cells were transient, and the levels of PD-1 expression were consistently restored to baseline levels within 3 days after each RMD dose.

### Polyfunctionality of HIVconsv-Specific Responses Increases After MVA_1_ and Decreases Over RMD Treatment

**Figure 5:**
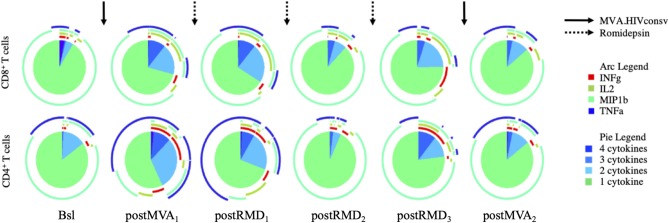
Longitudinal assessment of the T-cell cytokine production over the course of the intervention. Polyfunctionality of CD8^+^ and CD4^+^ T cells was analyzed by Boolean gating. Pie charts illustrate relative proportion of each of the different subsets (cells producing 1, 2, 3, or 4 cytokines, respectively).

Considering the effect of RMD on the expression of markers of cell death, exhaustion and activation, we assessed the effect of RMD on the functionality of vaccine-elicited T cells. Polyfunctionality of HIVconsv-specific T cells was measured by stimulation of PBMC with the HIVconsv peptides and enumeration of the cells producing IFN-γ, IL-2, MIP1-β, and/or TNF-α by flow cytometry. As shown in Figure 5, MVA_1_ vaccination increased relative polyfunctionality in both CD4^+^ and CD8^+^ HIVconsv-specific T cells. Three weeks after MVA_1_ (postMVA_1_), the highest increase in polyfunctionality was observed in vaccine immunogen-specific CD4^+^ T cells that produced 2, 3, and 4 cytokines (post MVA_1_, Figure 5), with a particular increase in INF-γ secreting cells. On the other hand, vaccine-specific CD8^+^ T cells produced INF-γ, IL-2, and TNF-α and were mostly polyfunctional, producing 2 or 3 cytokines. However, during the treatment with RMD, the polyfunctionality was reduced to baseline levels in both HIVconsv-specific CD4^+^ and CD8^+^ T cells and the final MVA_2_ vaccination was not able to re-boost functionality profiles (post MVA_2_, Figure 5 and Supplementary Figure 5). These results suggest that RMD treatment might have impaired functionality of vaccine-induced responses and prevented their subsequent booster vaccination effect.

### CD8^+^ Cells Maintain Antiviral Activity of After the RMD Treatment

**Figure 6:**
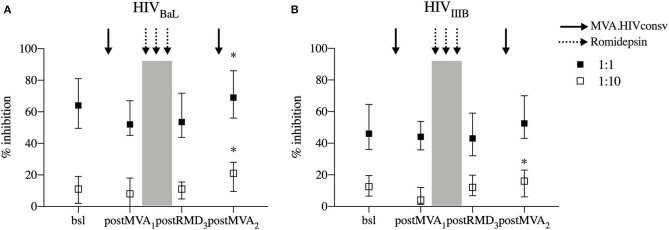
*In vitro* viral replication inhibition capacity. Levels of CD8^+^ viral inhibitory capacity are shown for HIV-1_BaL_ **(A)** and HIV-1_IIIB_ **(B)** at two E/T ratios (effector/target ratio) 1:1 and E/T 1:10 in individuals that underwent the intervention (*n* = 14 for HIV-1_BaL_ and *n* = 15 for HIV-1_IIIB_). *P*-values (*p < 0.05) are indicative for the corresponding timepoint compared with respective bsl.

To further study the functionality of vaccine-induced T cells during the intervention, we measured the *in vitro* antiviral capacity of CD8^+^ T cells. The *in vitro* replication inhibition capacity of PBMC-derived CD8^+^ T cells was measured by standard viral inhibition assay (VIA) (12, 31) using autologous CD4^+^ T cells infected with two laboratory-adapted HIV-1 strains BaL (R5 tropic virus) and IIIB (X4 tropic virus). Virus replication was measured by flow cytometry as the percentage of HIV-1 Gag p24-positive CD4^+^ cells. CD8^+^ T-cell inhibitory capacity was determined at screening, 3 weeks after MVA_1_ (postMVA_1_), 1 week after RMD_3_ (postRMD_3_), and 4 weeks after MVA_2_ (postMVA_2_). In contrast to the changes in cytokine production/polyfunctionality detected by flow cytometry, neither MVA_1_ nor RMD_1−2−3_ altered the inhibition activity, which was median of 64, 52, and 53,5% at the screening, postMVA_1_ and postRMD_3_ time points at the E:T ratio 1:1, respectively, against HIV-1_BaL_ and median 46, 44, and 43% at the screening, postMVA_1_ and postRMD_3_ time points, respectively, against HIV-1_IIIB_. In fact, there was a weak, but statistically significant increase in the inhibitory capacity against HIV-1_BaL_ after the second MVA vaccination given 4 weeks after RMD_3_ from median of 64% at baseline to 69% at postMVA_2_ at the E:T ratio 1:1 and from median of 11% at baseline to 21% at postMVA_2_, for the E:T ratio1:10 (Wilcoxon signed-rank, *p* = 0.0200) (Figure 6A) and against HIV-1_IIIB_ from median of 12% at baseline to 16% at postMVA_2_, for at the 1:10 E:T ratio (Wilcoxon signed-rank, *p* = 0.0156) (Figure 6B). These data indicate that antiviral activity, measured by VIA with laboratory-adapted viral strains, was not negatively impacted during RMD treatment.

## Discussion

The BCN02 study was an HIV-1 kick&kill proof-of-concept trial combining MVA.HIVconsv vaccines with the HDACi RMD given as an LRA to the previous ChAdV63.HIVconsv-MVA.HIVconsv regimen recipients from the parental BCN01 trial (12). The immune analyses in BCN02 showed a highly significant shift of HIV-1-specific T-cell responses toward conserved regions of HIV-1 covered by the immunogen and marked increases in histone acetylation and induction of viral transcription *in vivo* by RMD. However, the reduction of the viral reservoir after these interventions was overall minimal (27). Already low baseline levels of viral reservoir in the early-treated participants enrolled in the BCN02 study likely limited latency reversal activity of RMD and/or a potential toxicity of RMD (28, 32) on vaccine-induced T cells might have precluded the capability to observe a more pronounced reduction in the size of the viral reservoir. To address the latter possibility, we here evaluated the effect that the HDACi RMD had on the T-cell viability, activation and functionality throughout the intervention that included RMD given in a three weekly 5 mg/m^2^ BSA regimen.

Our data document a *in vivo* toxic effect of RMD on T cells 3 days after each RMD exposure. Although these effects were transient and viability of T cells was partially recovered 7 days after RMD_1_ and RMD_2_, there was an additive increase in apoptosis over the full RMD regimen, which was not fully restored by 7 days after RMD_3_. Jones et al. and Zhao et al. have described increases in cell death upon long term (18- and 72- h period) *in vitro* exposure to RMD, but no cytotoxic effect were observed when T cells were exposed to RMD for 4 h only, which is more similar to the *in vivo* regimen where the terminal half-life (*t*_1/2_) of RMD is estimated to be ~3 h (28, 32). Indeed, Clutton et al. exposed *ex vivo* PBMC to RMD for 3 h, washed them and cultivated them further in the absence of RMD for 3 days in order to mimic *in vivo* exposure, and reported that the cell viability was not changed after 3 or 24 h, but was significantly reduced at 48 and 72 h (33). These results concur with our observations showing a peak of apoptotic T cells 3 days after each RMD infusion and could explain the transient decreases in peripheral CD4^+^ T-cell counts observed in BCN02 (27). Also, the cumulative effect on viability observed after 3 RMD doses is in line with the delayed effects of RMD suggested by Clutton.

As RMD may induce T-cell activation resulting from reservoir reactivation and viral antigen presentation, which could also drive increase in exhaustion levels prior to the induction of apoptosis, we evaluated the frequency of activated CD4^+^ and CD8^+^ T cells. Indeed, the additive increase in HLA-DR^+^ activation markers in both CD8^+^ and CD4^+^ T cells over RMD treatment, especially in HIVconsv-specific T cells, was already observed after the first MVA.HIVconsv vaccination. Moreover, as the increased expression of HLA-DR was mostly observed in T effector memory cells and the fact that HIVconsv-specific T cells showed higher levels of HLA-DR expression, the data suggest that there could be a different memory distribution among HIVconsv-specific T cells compared to the total T cell population. These changes are in line with the increase in the magnitude of the immunogen-specific responses upon vaccination (27). However, the magnitudes of vaccine-specific responses were not increased further during RMD administration, suggesting that viral reactivation did not contribute in a major way to the increased levels of cell activation during RMD. In contrast, RMD treatment changed levels of CD69 and PD-1 more rapidly albeit transiently, peaking at 4 h after the end of each RMD dose. In line with our observation on CD69 longitudinal expression, an increase in CD69^+^ expression was previously described upon RMD, panobinostat and vorinostat treatments *in vitro* (18, 24) and also *in vivo* given alone (22) or in combination with a vaccine (34). This enhanced expression of PD-1 and CD69 could be the result of a generalized increased expression of the host genes induced by the action of RMD. So far, little is known about the effect of RMD and other LRA on the host genome gene expression profiles. There is an urgent need to better understand these effects and, especially, to clarify how they can affect the antiviral immunity against HIV-1 (35) or, in fact, fueling it.

Although it was previously described that RMD can impair T-cell and NK function *in vitro* (28, 29, 36, 37), Søgaard et al. presented *in vivo* data from the REDUC trial, indicating that RMD did not alter the proportion of HIV-1-specific T cells nor inhibited T-cell cytokine production (22), at least in the peripheral blood. Nonetheless, there appears to be a trend toward decreased HIV-specific T cell responses after RMD treatment, which may have not reached statistical significance due to the limited group size. On another note, vaccine-induced responses in the REDUC trial were weaker compared to the responses induced in the BCN02 trial (12, 27), possibly due to weaker vaccine vectors/immunogens employed in the REDUC trial. In addition, REDUC did not include early-treated individuals with relatively intact immunity, and the detection of reduced polyfunctionality of vaccine-induced T-cells could have been limited. In BCN02, the first MVA.HIVconsv vaccination enhanced polyfunctionality, especially in CD4^+^ T cells, in line with previous reports showing that MVA-vectored vaccination can improve the polyfunctionality and T effector memory phenotype in CD4^+^ T cells more so than in CD8^+^ T cells (38). This increased effector function profile induced by MVA_1_ was reduced upon RMD treatment but did not lead to a net reduction of the latent reservoir compared to baseline.

Our data also indicate that despite fluctuations in activation, maturation phenotypes and polyfunctionality, RMD treatment did not impair the *in vitro* antiviral capacity of CD8^+^ T cells. While some *in vitro* studies have shown a diminished inhibitory capacity when CD8^+^ T cells were exposed to RMD, but not to other HDACi (39, 40), such an effect was not observed *in vivo* in clinical trials using RMD. In the REDUC trial, the viral inhibition assay showed a trend toward increased inhibitory activity post-immunization that was lost after RMD exposure, but overall, antiviral capacity did not significantly change over time. Similarly, we did not see a reduction in the antiviral capacity of T cells over the course of RMD_1−2−3_, suggesting that the preservation of the antiviral capacity may depend on the balance between the deleterious effects of RMD and the potency of the employed vaccination strategy.

Of note, our results highlight that there is no direct comparability between assays used to characterize CTL functionality, including multiparametric flow cytometry and *in vitro* inhibition assays. In particular, in our study, the polyfunctionality of CD8^+^ T cells decreased slightly during RMD treatment, while *in vitro* VIA activity was maintained. In addition, the MVA_2_ boost vaccination did not augment the proportion of cell secreting multiple cytokines, while a moderately increased *in vitro* suppressive capacity was observed. These data are consistent with other studies, which showed a disconnection between cytokine secretion and antiviral capacity (41, 42), indicating that polyfunctionality of HIV-1-specific CD8^+^ T cells (at least as measured in standard assays) is not directly associated with viral suppression capacity (31, 43, 44). This lack of consistency between the two techniques could be due to the cytokines measured in standard protocols for intracellular cytokine assays: whilst IFN-γ, IL-2, MIP1-β, and TNF-α are used to measure functionality of HIV-1-specific T cells, evaluating secretion of granzyme B and perforin could be more accurate when assessing T-cell killing activity and may provide better concordance with VIA activity (45). However, whether antiviral capacity measured by standard *in vitro* VIA assay using laboratory-adapted viral strains will translate into effective *in vivo* killing or reactivated, HIV-1 infected cells remain to be determined as well. In addition, further work using autologous virus might be more representative of the physiological conditions in kick&kill strategies. Finally, viral suppression capacity was measured in total bulk CD8^+^ T cells while cytokine secretion was measured in vaccine specific cells, which could have been more susceptible to *in vivo* effects by RMD.

This present study has a number of limitations, which include a small sample size and the lack of control arms, both placebo and single intervention arms to discern the effects exerted by RMD or the vaccine alone. Thus, the presented results need to be interpreted with caution. Still, the present study shows that RMD has a transient effect on T-cell viability, exhaustion and increased cell activation in an additive way over three weekly doses. Although this can result in a decrease of polyfunctionality of vaccine-induced HIVconsv-specific responses, the *in vitro* replication inhibition capacity of CD8^+^ T cells was not impaired and should not preclude effective killing upon RMD-induced viral reactivation. As RMD increased higher levels of HIV-1 transcription on the RMD_2_ and RMD_3_ dose, it is tempting to speculate that increasing the number the RMD doses could result in further increased in levels of reservoir reactivation without overly inhibiting the antiviral capacity of CD8^+^ T cells. Regardless whether this would occur *in vivo* with longer term RMD administration, the present data indicate that timing and order of LRA and T-cell immunotherapy regimens are critical in order to achieve the clearance of reactivated latently HIV-1-infected cells. Larger controlled clinical trials are needed to further investigate combinations of LRA and immune intervention in order to find the best strategy to achieve a functional cure of HIV-1.

## Data Availability Statement

The datasets generated for this study are available on request to the corresponding author.

## Ethics Statement

This study was carried out in accordance with the recommendations of and approval by the Ethics Committee of the Hospital Universitari Germans Trias i Pujol (Badalona, Spain). All subjects provided their written informed consent to participate. The study was conducted according to the principles expressed in the Declaration of Helsinki.

## Author Contributions

MR-U, MR-R, BM, and CB conceived and designed the study and drafted the manuscript. MF, MM, PC, CM, SC, JMM, BC, TH, and JM contributed to the study design. MR-U, MR-R, and MF performed the experiments. All authors revised the manuscript critically for important intellectual content and approved the final version of the manuscript.

### Conflict of Interest

JMM reports grants and personal fees from Abbvie, Angelini, Contrafect, Genentech, Gilead, Jansen, Medtronic, MSD, Pfizer, ViiV Healthcare, outside the submitted work. TH reports grants from Medical Research Council UK, during the conduct of the study, and has a patent US 7981430B2 issued. CB is founder, CSO and shareholder of AELIX THERAPEUTIC, S.L. BM is a consultant for AELIX THERAPEUTICS, S.L., outside the submitted work. The remaining authors declare that the research was conducted in the absence of any commercial or financial relationships that could be construed as a potential conflict of interest.
