# Supplementary material for: In vivo Effects of Romidepsin on T-Cell Activation, Apoptosis and Function in the BCN02 HIV-1 Kick&Kill Clinical Trial
Source: Front Immunol. 2020 Mar 20;11:418. doi: 10.3389/fimmu.2020.00418 (PMC7100631; doi:10.3389/fimmu.2020.00418)
Supplement: Supplementary file 1 [file Presentation_1.pptx]

## Slide 1
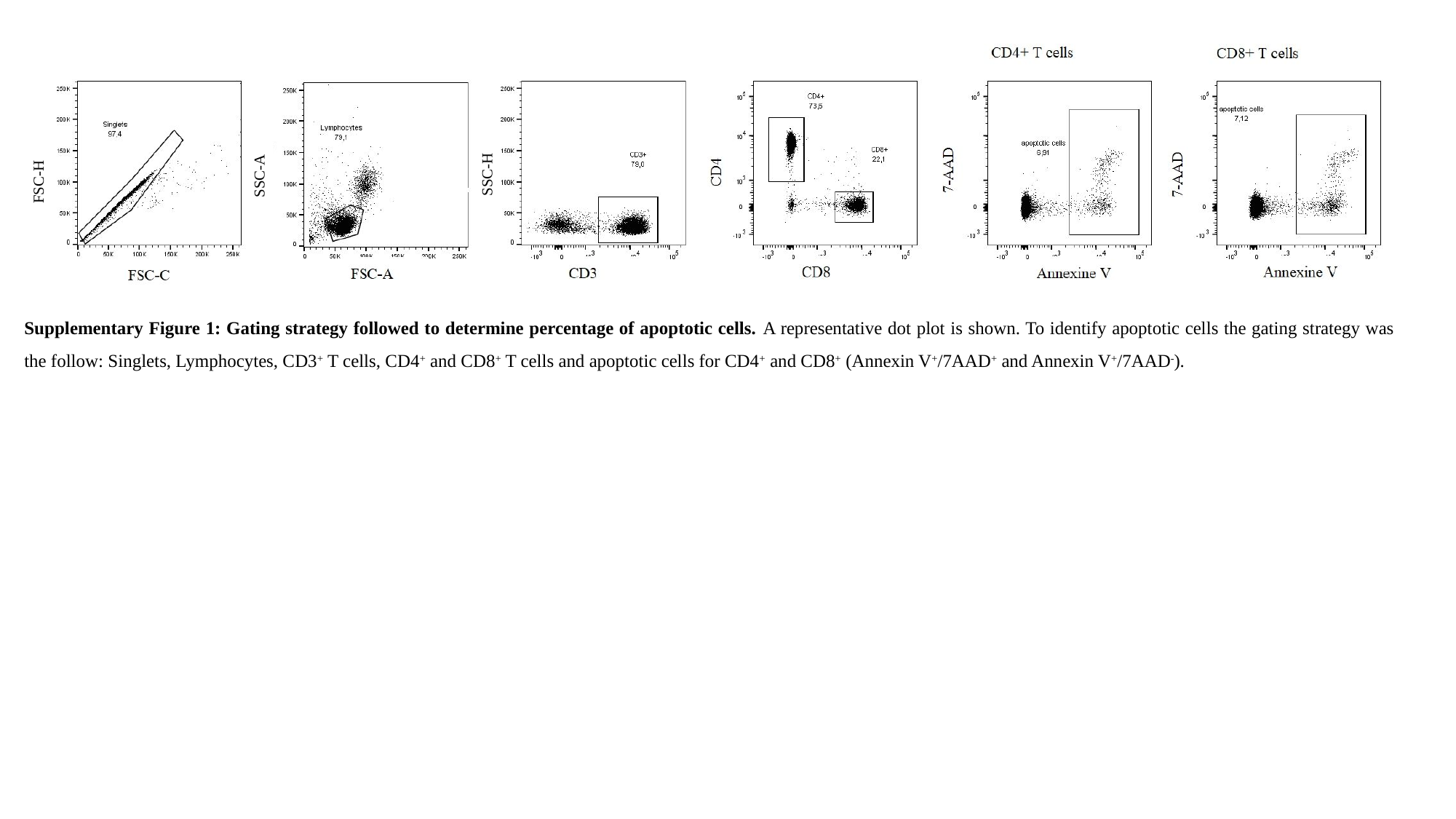

SSC-H
SSC-A
FSC-H
SSC-A
FSC-H
Supplementary Figure 1: Gating strategy followed to determine percentage of apoptotic cells. A representative dot plot is shown. To identify apoptotic cells the gating strategy was the follow: Singlets, Lymphocytes, CD3+ T cells, CD4+ and CD8+ T cells and apoptotic cells for CD4+ and CD8+ (Annexin V+/7AAD+ and Annexin V+/7AAD-).

## Slide 2
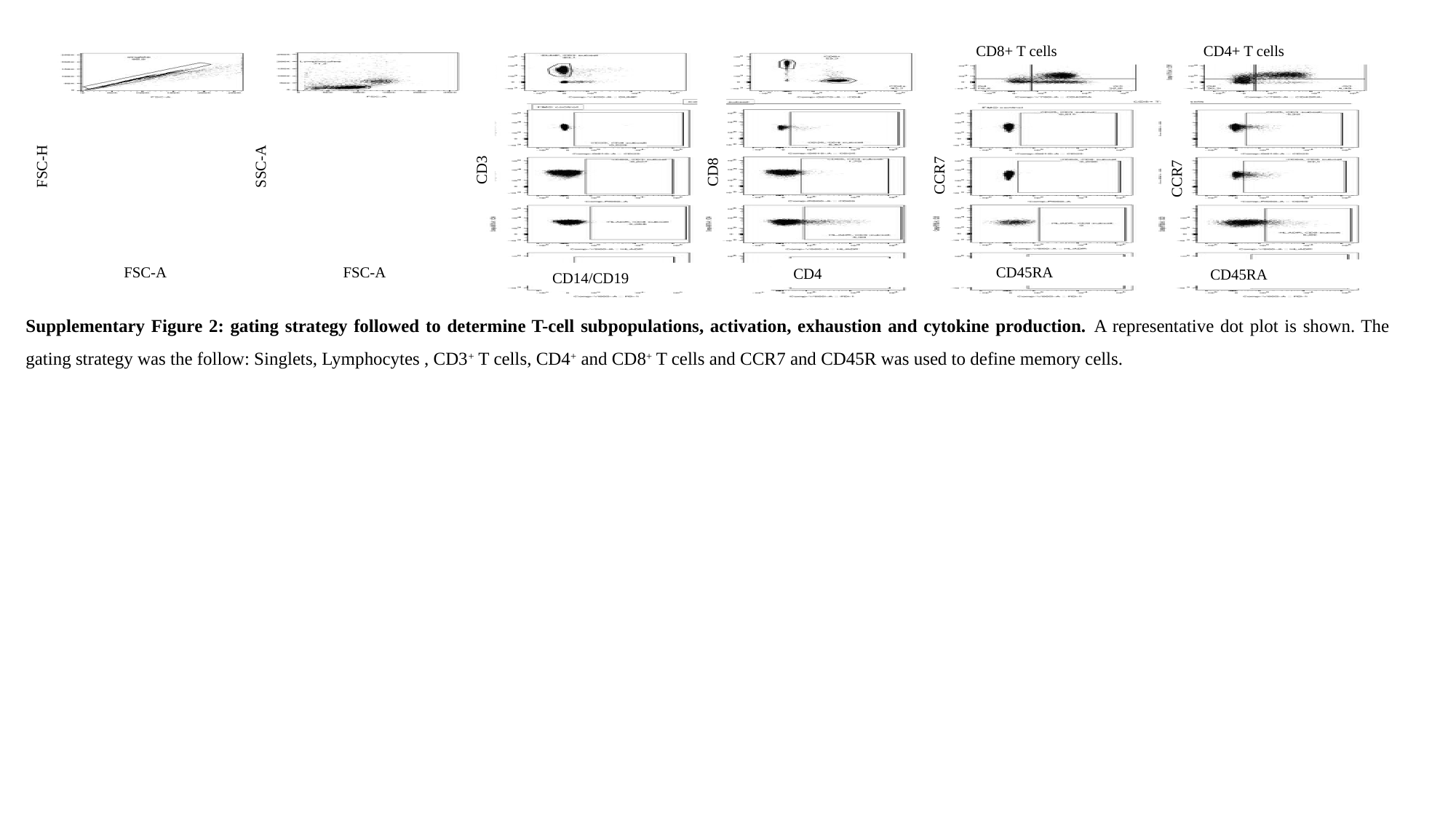

CD8+ T cells
 CD4+ T cells
 CD3
 CD8
 CCR7
 CCR7
CD45RA
FSC-A
FSC-A
 CD4
CD45RA
CD14/CD19
 FSC-H
 SSC-A
Supplementary Figure 2: gating strategy followed to determine T-cell subpopulations, activation, exhaustion and cytokine production. A representative dot plot is shown. The gating strategy was the follow: Singlets, Lymphocytes , CD3+ T cells, CD4+ and CD8+ T cells and CCR7 and CD45R was used to define memory cells.

## Slide 3
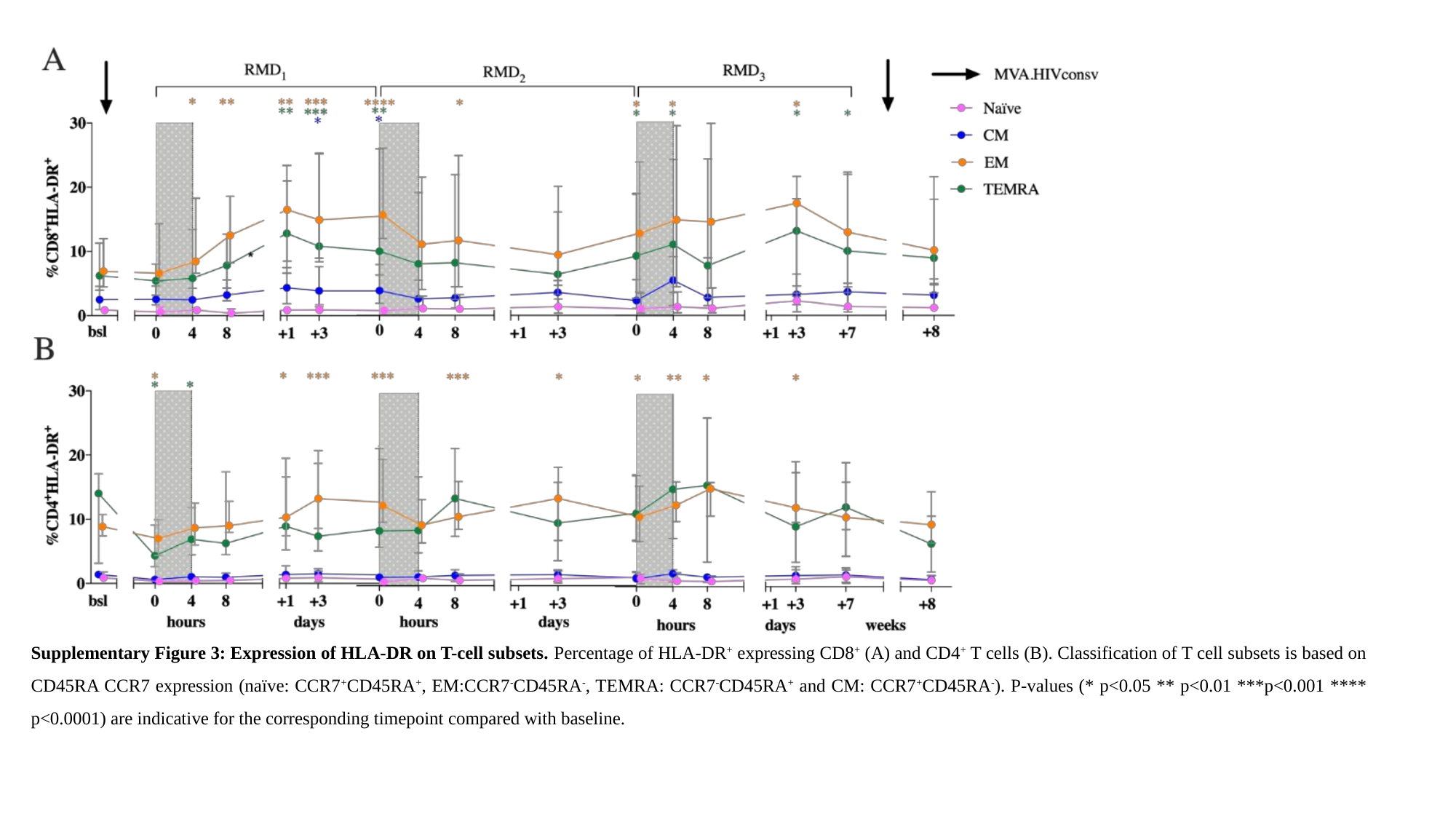

Supplementary Figure 3: Expression of HLA-DR on T-cell subsets. Percentage of HLA-DR+ expressing CD8+ (A) and CD4+ T cells (B). Classification of T cell subsets is based on CD45RA CCR7 expression (naïve: CCR7+CD45RA+, EM:CCR7-CD45RA-, TEMRA: CCR7-CD45RA+ and CM: CCR7+CD45RA-). P-values (* p<0.05 ** p<0.01 ***p<0.001 **** p<0.0001) are indicative for the corresponding timepoint compared with baseline.

## Slide 4
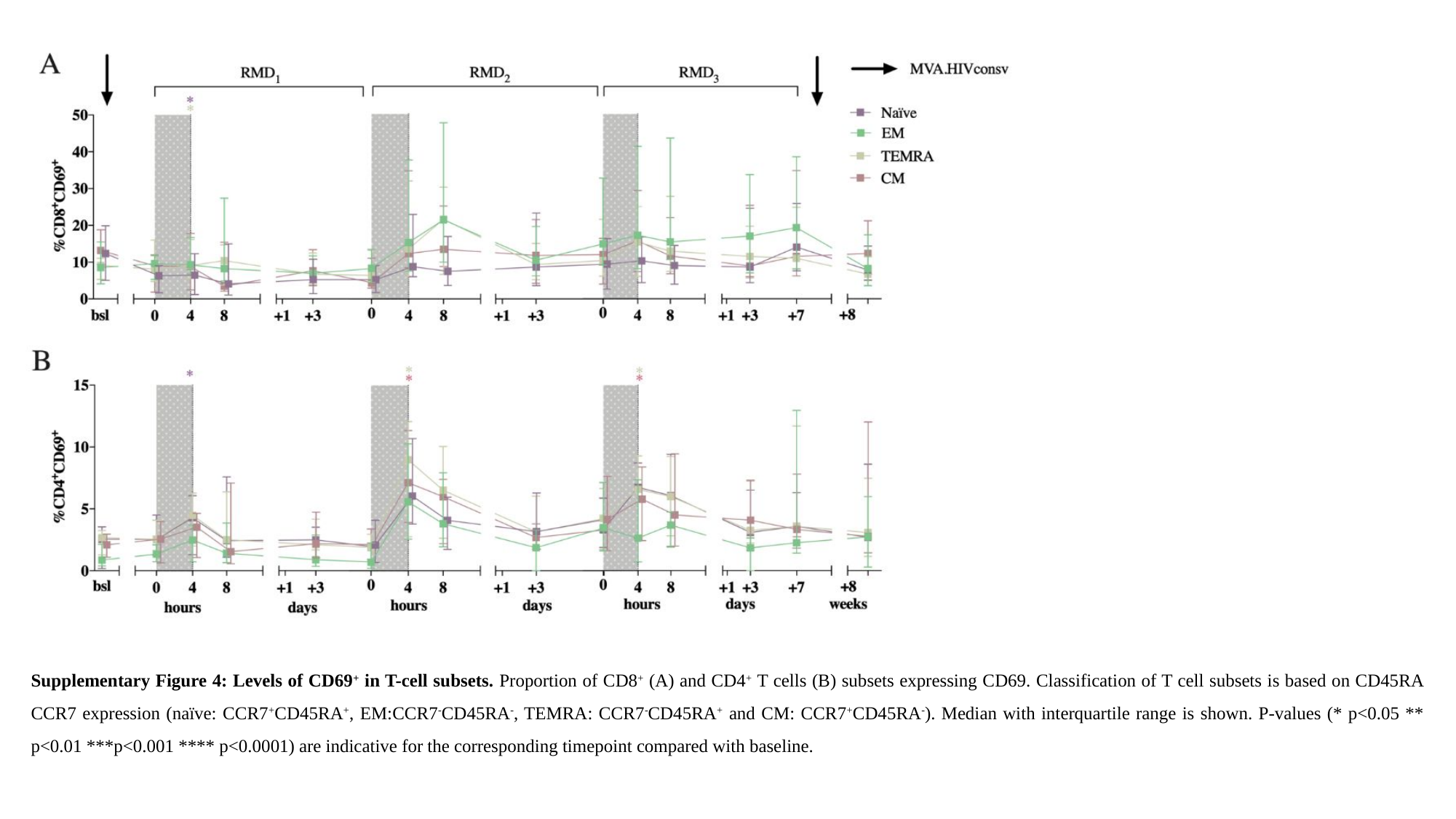

Supplementary Figure 4: Levels of CD69+ in T-cell subsets. Proportion of CD8+ (A) and CD4+ T cells (B) subsets expressing CD69. Classification of T cell subsets is based on CD45RA CCR7 expression (naïve: CCR7+CD45RA+, EM:CCR7-CD45RA-, TEMRA: CCR7-CD45RA+ and CM: CCR7+CD45RA-). Median with interquartile range is shown. P-values (* p<0.05 ** p<0.01 ***p<0.001 **** p<0.0001) are indicative for the corresponding timepoint compared with baseline.

## Slide 5
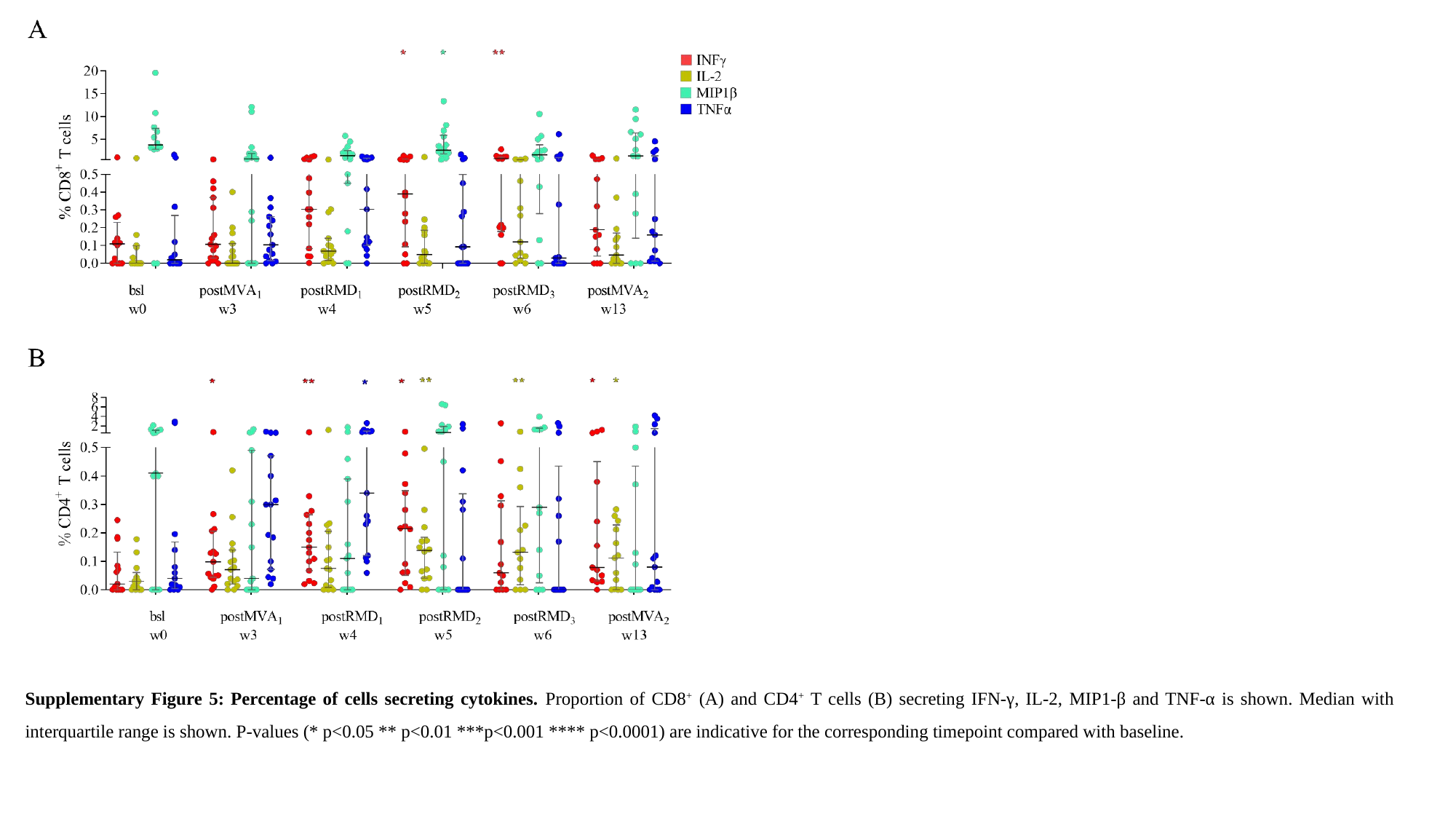

Supplementary Figure 5: Percentage of cells secreting cytokines. Proportion of CD8+ (A) and CD4+ T cells (B) secreting IFN-γ, IL-2, MIP1-β and TNF-α is shown. Median with interquartile range is shown. P-values (* p<0.05 ** p<0.01 ***p<0.001 **** p<0.0001) are indicative for the corresponding timepoint compared with baseline.
